# Monitoring of water quality with HPLSEC and fluorescence method in the ozonated recirculating aquaculture system

**DOI:** 10.1007/s10661-023-12117-5

**Published:** 2023-11-20

**Authors:** Samu Pettersson, Alexey Ignatev, Petra Lindholm-Lehto, Tuula Tuhkanen

**Affiliations:** 1 Department of Biological and Environmental Sciences, University of Jyväskylä, Survontie 9C (Ambiotica), 40500 Jyväskylä, Finland; 2 Aquatic Production Systems, Natural Resources Institute Finland (Luke), Survontie 9A, 40500 Jyväskylä, Finland

**Keywords:** Advanced oxidation process, Fluorescence, High-performance size exclusion chromatography, Hydrogen peroxide, Natural organic matter, Oxidative treatment

## Abstract

In recirculating aquaculture systems (RAS), it is important to monitor the water quality to keep the fish healthy. Especially in water treatment with oxidizing agents, for example, ozone (O_3_) or hydrogen peroxide (H_2_O_2_), the monitoring of the quality of dissolved organic matter (DOM) in water is advisable to keep track of the treatment’s effect. Previously in wastewater treatment plant (WWTP) studied, HPLSEC and fluorescence method for monitoring and characterization of organic matter was used here to track the effect of oxidative treatments; 5 duplicated treatments (2 × O_3_, 1 × O_3_ + H_2_O_2_, 1 × H_2_O_2_, 1 × control) were performed for four months with weekly samplings. Systems that contained O_3_ injection reduced fluorescence on average over 90%, except tyrosine-like fluorescence with removal of 80%. Combined O_3_ + H_2_O_2_ treatment did not bring any advantages over pure O_3_ treatment, and H_2_O_2_ had no significant effect on fluorescence. Humic and fulvic compounds were detected to largely be derived from inlet lake water, while large protein-like structures were mostly created in RAS. A peak of benzoic acid-like molecules was also detected in all RAS waters. Treatments did not change the molecular weight profile of DOM systems and inlet water, having most of their fluorescence coming from medium-sized (108–1322 Da) molecules. DOC was lower in O_3_ treatments, but the linear connection between DOC and fluorescence was not observed, although this was proposed in earlier studies. Most likely, the oxidizing treatments induce change to DOM in a way that such comparison becomes inaccurate, although this must be studied further in the future.

**Supplementary Information:**

The online version contains supplementary material available at 10.1007/s10661-023-12117-5.

## Introduction

Ozone is a strong organic oxidizer, widely used nowadays in industry and domestic water production due to the ozone’s ability to oxidize most of the organic material. This ability has been noted in the aquacultural industry since ozone can be used to increase fish welfare by disinfecting pathogens and improving overall water quality (Summerfelt et al., 1997; Powell, 2016; Davidson et al., 2021). Ozone also has the potential to remove problematic off-flavor compounds from water that is especially abundant in recirculating aquaculture systems (RAS) (Lindholm-Lehto & Vielma, 2019; Pettersson et al., 2022). Even though ozone rapidly decomposes to oxygen, it is still very toxic to fish, and this raises concern among fish farmers, although the research and usage have steeply risen during the last few decades (Powell, 2016). To mitigate risks and costs, it is important to optimize and monitor the ozone’s performance. One possible way for monitoring is to use high-performance liquid size exclusion chromatography (HPLSEC) with fluorescence and UV detection to track the decomposition of dissolved organic matter (DOM).

Currently, DOM in aquaculture water is studied frequently in multiple different ways. PARAFAC method (one of the most used ones) was used, for example, by Hambly et al. (2015), Yamin et al. (2017), and Kim et al. (2021). These studies have a comprehensive evaluation of fluorescence data, but molecular weights were not studied. Kamjunke et al. (2017) did an extensive evaluation of DOM in aquacultural water with nuclear magnetic resonance spectroscopy, ultrahigh-resolution spectrometry, and fluorescence, especially with tyrosine- and tryptophan-like fluorescence. Aguilar-Alarcon et al. (2020, 2022) used high-resolution and time-of-flight mass spectrometry to evaluate DOM in RAS without fluorescence. The latter study focuses especially on fulvic acids in DOM. Wang et al. (2021) used PARAFAC and cyclotron resonance mass spectrometry to identify DOM from aquacultural ponds.

The usage of HPLSEC and fluorescence in water purification has been previously studied by Ignatev and Tuhkanen (2019). They concluded that it is indeed viable to monitor the change of organic matter during the wastewater purification process with the HPLSEC method. Other studies have also found this method viable for monitoring water treatment processes (Hidayah et al., 2020; Jokubauskaite et al., 2015). Spiliotopoulou et al. (2017, 2018) used fluorescence and HPLSEC in a marine water aquarium system and small RAS to successfully monitor the effect of ozone on the quality of DOM, but other than that, the application of HPLSEC and fluorescence to ozonated RAS to monitor DOM is largely unstudied.

This study is the second part of an earlier study (Pettersson et al., 2022) about the use of oxidizers in removing the off-flavors from RAS and included the off-flavor and fish data (Pettersson et al., 2022). On the other hand, this study contains the characterization of the amount and size distribution of DOM. The aim of this part is to study the viability of the fluorescence and HPLSEC method for RAS water and the change of organic matter due to the ozonation. According to our knowledge, this is the first time when organic matter in continuously ozonated RAS was studied and regularly with different ozone and hydrogen peroxide amounts. It was hypothesized that the more intensive the oxidizing treatment, the lower the fluorescence and UVA-254 would be. With the development of monitoring methods and tools, it is possible to encourage the aquacultural industry to wider use of ozone. Correctly, ozone addition should reduce maintenance costs, keep disease outbreaks under control, and support the growth of the fish, which finally leads to economic savings.

## Materials and methods

### Experimental setup

This study is the second part of our earlier study (Pettersson et al., 2022), and the data in this article was collected at the same time. A more detailed description of ozonation equipment, experiment conditions, and sampling can be found in the aforementioned article.

In short, the 10 individual RAS contained a bottom-drained rearing tank, feed collector, solids removal (vortex clarifier and a drum filter with 60-µm mesh size), a moving-bed biofilter, a forced-ventilated cascade aeration column for CO_2_ removal, oxygen injection, and pH adjustment with NaOH solution. The total volume of the system was 1440 L. At the beginning of the experiment, each system contained approximately 23.8 kg of rainbow trout (*Oncorhynchus mykiss)*. The water renewal rate was 5.4–6.0 L h^−1^ and was adjusted based on feeding (500 L of water per kg of feed per day). Feeding ratio changed between 0.9 and 1.2% during the experiment. The system pH was maintained at 7. All the systems were maintained identically, excluding the different oxidizing treatments. The experiment lasted for four months (Pettersson et al., 2022).

The experiment included treatments: low O_3_ (0.4 mg L^−1^), high O_3_ (0.8 mg L^−1^), O_3_ + H_2_O_2_ as advanced oxidation process (0.4 mg L^−1^ and 0.15 µl L^−1^), H_2_O_2_ (0.15 µl L^−1^), and control (*n* = 2, 10 systems in total). The lake water from Lake Peurunka (62.44886, 25.85201, 694 ha, 59,600 m^3^) was used as new replacement water and was also studied during the experiment (inlet lake water). The flow in the ozonation loop was 0.23 L s^−1^.

### Sampling

The experiment lasted for four months. Samples were collected weekly from the rearing tank below the surface with an empty plastic syringe. Samples were immediately filtered through 0,45-μm prewashed cellulose acetate syringe filters (Sartorius, 16555-Q) to 50-ml sample tubes made of polypropylene and high-density polyethylene (VWR) and stored in a fridge (at 6 °C) for few days before the analysis.

### Chromatography and fluorescence method

The HPLSEC method was based on a method described by Ignatev and Tuhkanen (2019). The mobile phase was prepared for the analysis by dissolving 0.8900 g of Na_2_HPO_4_ (analytic grade, WVR Chemicals) and 0.7801 g of NaH_2_PO_4_ (analytic grade, WVR Chemicals) to 2 L of ultrapure water. The solution was then vacuum filtered through a Whatman 0.2-µm cellulose acetate filter and transferred to an analyzer. A stirred sample (1.5 mL) was transferred to the HPLSEC-glass vial (2 ml Verex vial, Phenomenex). HPLSEC analysis was performed with C196-E061W Prominence (Shimadzu, Japan) measuring tryptophan-, tyrosine-, fulvic-, and humic-like fluorescence and UV absorbance at 254 nm. A separation column (Yarra 3 μm SEC-3000, 300 * 7.8 mm, Phenomenex, USA) was used. Each sample was analyzed twice with two different wavelengths for fluorescence. The used wavelengths were 270 nm excitation and 355 nm emission for tryptophan, 220 nm excitation and 310 nm emission for tyrosine. Humic-like fluorescence was followed at 330 nm excitation and 425 nm for emission, and fulvic had 390 nm excitation and 500 nm for emission. After every three samples, a blank sample (ultrapure water) was run to check that no impurities were present. The fluorescence intensity was integrated into Shimadzu LabSolutions LC/GC (version 5.51.) for each different wavelength.

**Fig. 1:**
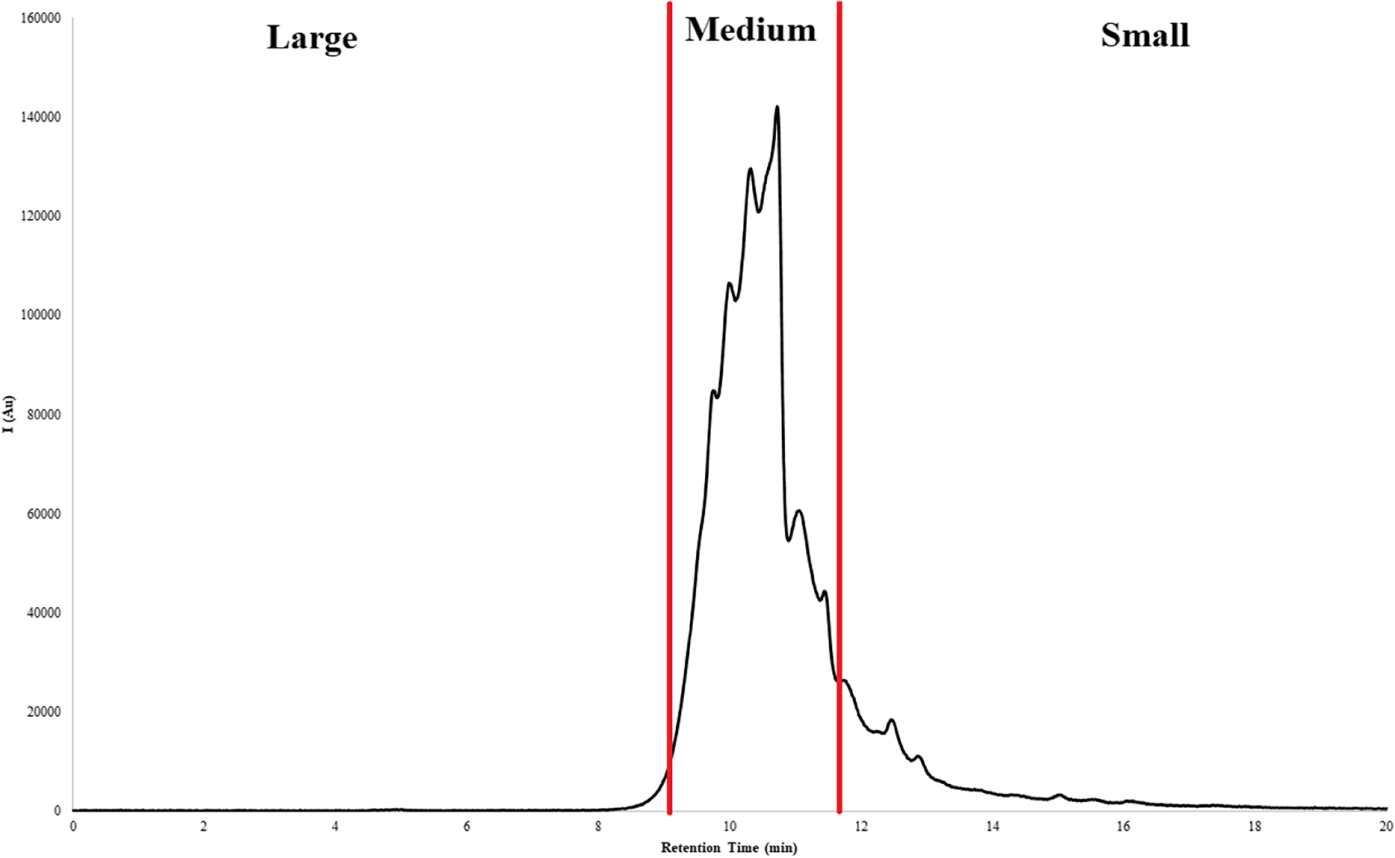
For example, initial humic-like fluorescence between retention time of 0–20 min from system 1 showing the partition to different size fractions (large, medium, and small)

Fluorescence data was separated into three fractions according to the main bulk of fluorescence intensity. These fractions were as follows (retention time in brackets): large (0.0–9.0 min), medium (9.0–11.8 min), and small (11.8–20 min) (Fig. 1). Fluorescence area integration was performed manually for size fractions. It was calculated that the percentage of one fraction on average contributed to fluorescence and the UVA-254 during the experiment. MS Excel 2016 (v16.0) in Office 2016 (Microsoft Corp. 2015) was used for handling the fraction data.

Standards were run to link the retention times to molecular weights, and a standard curve of *R*^2^ = 0.992 was acquired with good logarithmic linearity (Supplementary Fig. 1). The lowest and the highest atomic weights of size fractions are shown in Table 1.

**Table 1:** The lowest (low) and highest (high) atomic weights (Da) for all size fractions and corresponding retention times (“t start” and “t end”)

Size fraction	*t* start (min)	*t* end (min)	High (Da)	Low (Da)
Large	0	9	4205031	1322
Medium	9	11.8	1322	108
Small	11.8	15	108	6

### Statistical analyses

Data analyses were performed using Microsoft Excel (2016) and IBM SPSS statistics 24. Kruskal–Wallis’ test (significance level 0.05) was used to test the average fluorescence difference between treatments after week 3. The same Kruskal–Wallis test was also used to compare size fractions between the treatments and inlet lake water. DOC concentrations were compared between treatments with Friedmann’s test (significance level 0.05). The DOC-fluorescence relationship was presented with a general linear model, and the *R*^2^ value was determined for models by taking DOC data from the systems and plotting it against fluorescence and UVA-254 separately.

Ozone doses were calculated as mg of injected ozone per mg of DOC in a liter of water. This was calculated for the ozonation loop and its water flow rate. It was assumed that all of the injected ozone were consumed in the process. This was calculated for every week as DOC concentrations changed through the experiment.

## Results

### Reduction of DOC

**Fig. 2:**
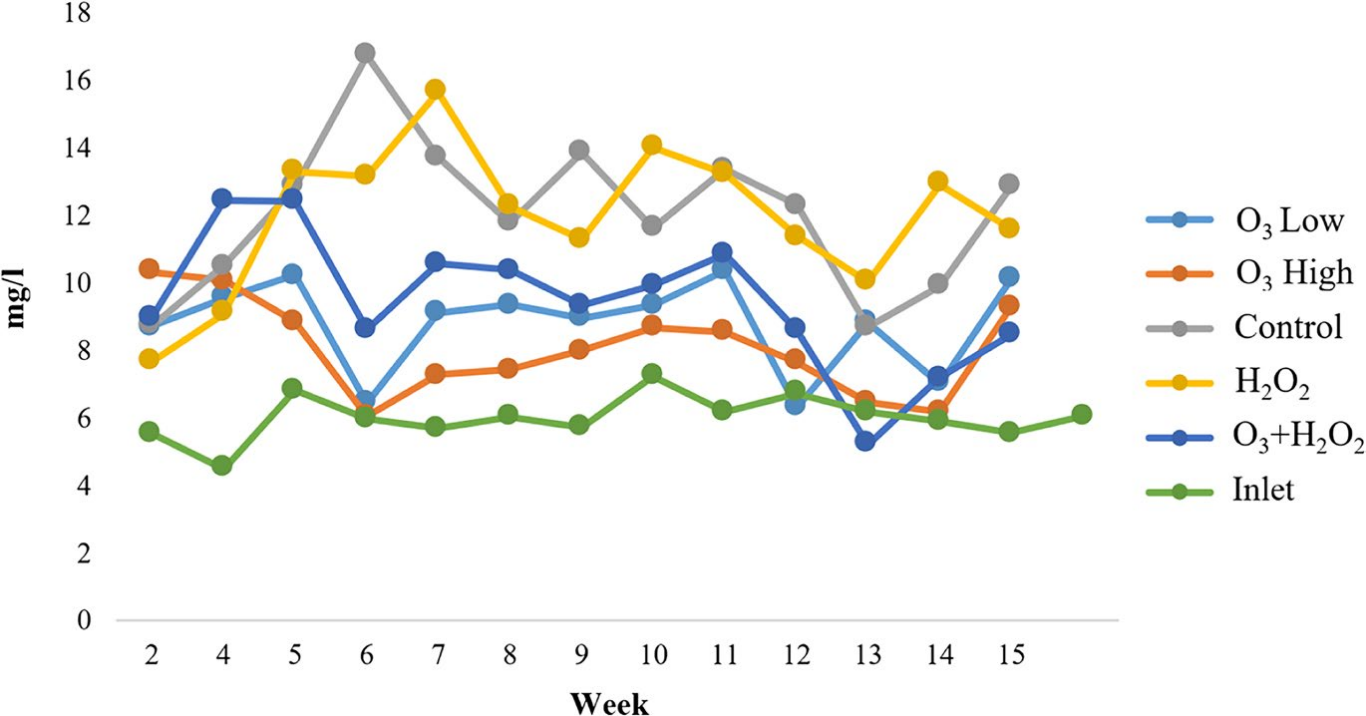
DOC concentrations (mg L.^−1^) during the experiment for all treatments and inlet lake water (LW)

The DOC concentrations were lower in ozone-treated systems than in the control and H_2_O_2_ systems (*n* = 28, *F* = 4, *p* < 0.05), and the high O_3_ had the lowest DOC values. High O_3_ had statistically lower values than O_3_ + H_2_O_2_ (*n* = 28, *F* = 4, *p* < 0.05). Hydrogen peroxide did not seem to have any noticeable effect on DOC concentrations when compared to controls (*n* = 28, *F* = 4, *p* = 0.866) (Supplementary Table 2). The measured DOC concentrations were approximately 3–4 mg L^−1^ lower in low O_3_ and O_3_ + H_2_O_2_ treatments than in control and H_2_O_2_ (Fig. 2). High O_3_ systems had over 5 mg L^−1^ lower DOC concentrations than control and H_2_O_2_, closing similar values with inlet water (Fig. 2).

Ozone dose in high O_3_ systems was about two times higher than in O_3_ + H_2_O_2_ and low O_3_ systems. The dose in the latter two stayed similar throughout the experiment. Fluctuations and few peaks were recorded in all treatments, especially in weeks 6 and 12–14. The average dose was 0.106 mg (O_3_) mg^−1^ (DOC) for high O_3_, 0.047 mg (O_3_) mg^−1^ (DOC) for low O_3_, and 0.044 mg (O_3_) mg^−1^ (DOC) in O_3_ + H_2_O_2_ (Supplementary Fig. 2).

### Fluorescence and UVA-254

**Fig. 3:**
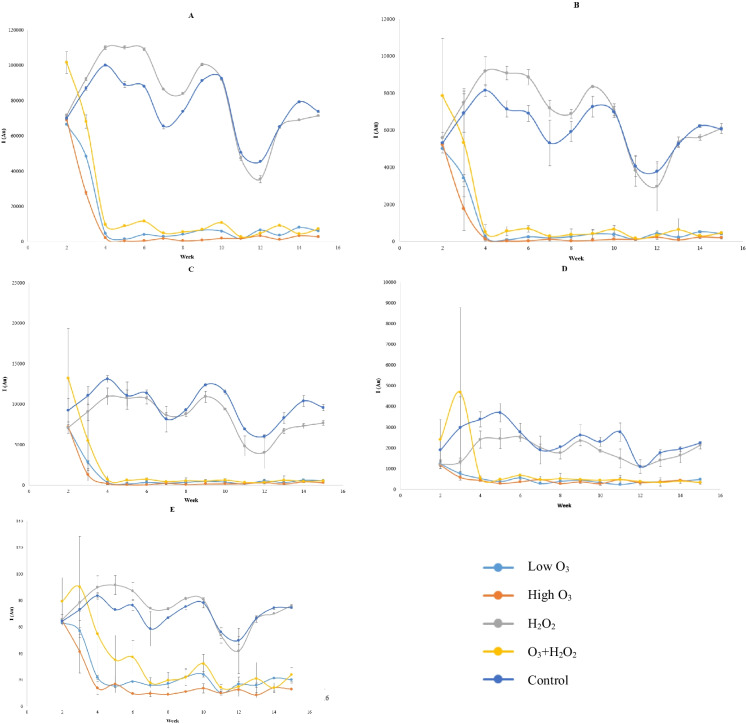
Averaged fluorescences (**A** = humic, **B** = fulvic, **C** = tryptophan, **D** = tyrosine) and UV-254 (= **E**) (*n* = 2, ± SD) during the experiment in all treatments. *Y*-axis displays the intensity (I) as absorbance units (AU)

Humic-like fluorescence was by far the most abundant of all the measured wavelengths, followed by tryptophan-, fulvic-, and tyrosine-like fluorescence. All the treatments, except H_2_O_2_, managed to remove the majority of the fluorescence from RAS during the first two weeks, after which the values stayed at similar levels (Fig. 3).

Treatments that included ozone removed 90–96% of fulvic-, humic-, and tryptophan-like fluorescence. High O_3_ had slightly better fluorescence removal performance than low O_3_, only by a few percent, but statistically, there was a significant difference in humic (*n* = 132, *F* = 5, *p* < 0.05) and fulvic fluorescence (*n* = 132, *F* = 5, *p* < 0.05), but with tryptophan, no statistical difference was found (*n* = 132, *F* = 5, *p* = 0.086). When compared to O_3_ + H_2_O_2_ treatment, high O_3_ dose had consistently lower fluorescence (*n* = 132, *F* = 5, *p* < 0.05) in all previously mentioned cases. There was no statistical difference between O_3_ + H_2_O_2_ and low O_3_ treatments (*n* = 132, *F* = 5, *p* > 0.05), although O_3_ + H_2_O_2_ performance seemed to be 2–5% percent lower overall. All these three ozone treatments had statistically lower fluorescence values than controls and H_2_O_2_ treatments (*n* = 132, *F* = 5, *p* < 0.05). No difference was recorded between H_2_O_2_ and controls (*n* = 132, *F* = 5, *p* > 0.05) for fluorescence or UVA-254.

Ozonated treatments removed tyrosine-like fluorescence with efficiency of 80–84% (Table 2). There was no statistical difference between them (*n* = 132, *F* = 5, *p* > 0.05), but a clear difference to controls and H_2_O_2_ treatment was recorded (*n* = 132, *F* = 5, *p* < 0.05) (Supplementary Table 1).

**Table 2:** Average percentual removal (+ SD) of fluorescence and UV-254 from weeks 4–15 when compared to the control treatment

	Humic	Fulvic	Tryptophan	Tyrosin	UVA-254
O_3_ low	93.7 ± 3.2	95.1 ± 2.6	95.9 ± 1.9	82.2 ± 5.8	73.7 ± 4.0
O_3_ high	97.6 ± 1.8	98.0 ± 1.6	97.8 ± 1.5	83.5 ± 5.8	82.8 ± 3.7
H_2_O_2_	5.7 ± 15.5	8.5 ± 16.2	15.5 ± 11.5	16.2 ± 41.2	− 5.9 ± 11.9
O_3_ + H_2_O_2_	90.7 ± 2.6	92.7 ± 2.2	94.6 ± 0.8	79.7 ± 5.6	64.2 ± 12.3

UVA-254 was removed less efficiently when compared to fluorescence (Table 2). Treatment with pure H_2_O_2_ failed to reduce it at all (*n* = 132, *F* = 5, *p* > 0.05), while the removal efficiency in other treatments ranged from 64 to 83%. High O_3_ had the highest removal efficiency with 83%. There was no statistical difference between O_3_ + H_2_O_2_ and low O_3_, although the first had UVA-254 mean removal efficiency of 64% and the latter 74% (Table 2).

In many cases, low O_3_, high O_3_, and O_3_ + H_2_O_2_ treatments were able to reduce fluorescence and UV-254 lower than in incoming inlet water, excluding tyrosine fluorescence, where the values were in the same range.

**Fig. 4:**
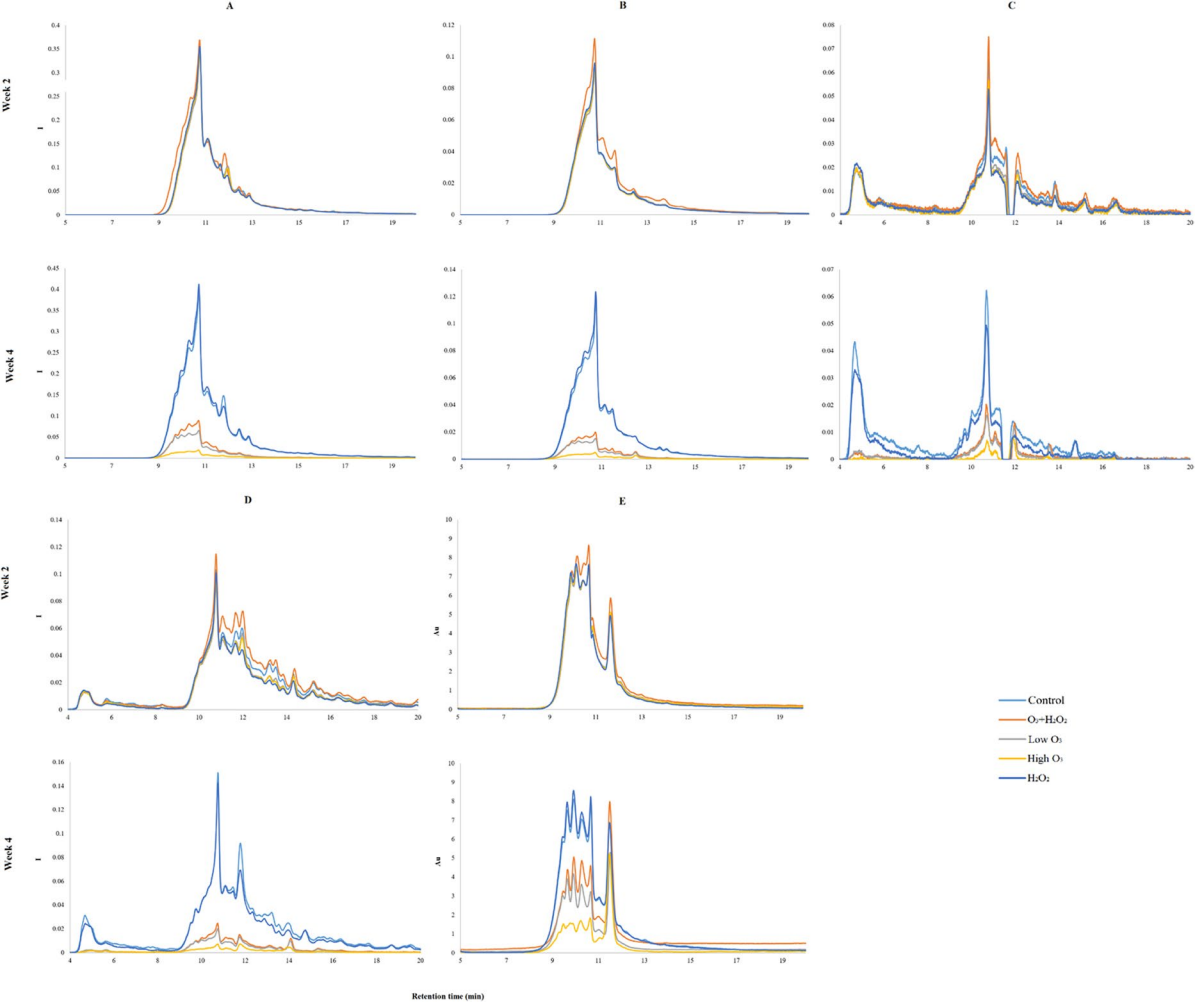
Averaged chromatograms for all treatments (orange = O_3_ + H_2_O_2_, gray = low O_3_, yellow = high O_3_, blue = H_2_O_2_, light blue = control) and fluorescence including UV-254 (**A** = humic, **B** = fulvic, **C** = tyrosine, **D** = tryptophan, **E** = UV-254). The *X*-axis displays the measured retention time (min), and the *Y*-axis displays the intensity (I) as absorbance units (AU). Chosen weeks for display were initial week 2 and week 4 when the treatments had stabilized

The main fluorescence peak was found around 11 min of retention time. Especially humic and fulvic fluorescences were largely defined by this peak, whereas tyrosine and tryptophan fluorescence contained a few other important ones in addition. The peak found around 5 min was exclusive only to these protein-like wavelengths, especially strong in tyrosine-like fluorescence. Tryptophane-like fluorescence also had a peak in 12 min, but this was more strongly present in control and H_2_O_2_ treatments during week 4 rather than 2 (Fig. 4). This peak was also missing from inlet lake water chromatograms (Supplementary Fig. 4).

In general, treatments of high O_3_, low O_3_, and O_3_ + H_2_O_2_ were able to reduce the fluorescence substantially when comparing chromatograms from the initial week 2 to week 4. However, H_2_O_2_ treatment did not have any effect on chromatograms and resembled closely controlled chromatograms, sometimes demonstrating slightly lower main peaks (for example, tyrosine, 5 min and 11 min). The chromatogram signals seemed to follow the following pattern in week 4 from lowest signal to highest: high O_3_, low O_3_, O_3_ + H_2_O_2_, and shared fourth position for H_2_O_2_ and control (Fig. 4).

Tyrosine-like fluorescence had the most erratic chromatograms and displayed the effect of treatments the least clearly. However, clear fluorescence reduction can be seen in peaks around 5 min and 9 min with high O_3-_, low O_3-_, and O_3_ + H_2_O_2_ treatments (Fig. 4). Tyrosine signal was substantially weaker and clearer in inlet lake water chromatograms when compared to any other RAS chromatograms (Supplementary Fig. 4).

UVA-254 also had initially during week 2 its core intensity found at 9 to 11 min of retention time, but it was spread more evenly. Another slightly weaker peak was found again around 12 min (Fig. 4). Inlet lake water lacked this peak almost completely (Supplementary Fig. 4). The even spread of UVA-254, however, changed in week 4 as between 9 and 11 min; four strong peaks can be easily distinguished in all treatments except high O_3_. High O_3_ treatment smoothed the peaks so that possibly only two can be observed. The peak in 12 min also seemed to grow in relation to other peaks, especially in O_3_-treated systems (for example, peak 12 min is many times higher than peaks around 9–11 min in high O_3_ treatment). Interestingly, O_3_ + H_2_O_2_ had the strongest fluorescence intensity this time during week 4, even higher than controls or H_2_O_2_. Otherwise, UVA reduction happened in a similar way as previously mentioned for fluorescence (Fig. 4).

### Molecular size fractions

**Fig. 5:**
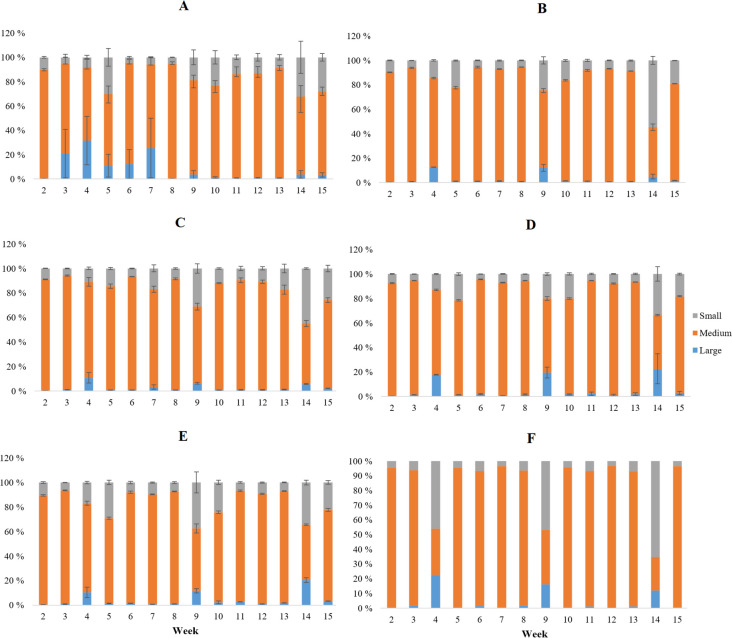
Molecular percentage for size fractions (large, medium, small) with standard deviations (excluding inlet lake water) from the start of the experiment (week 2) to the end (week 15). Treatments were O_3_ + H_2_O_2_ (**A**), low O_3_ (**B**), high O_3_ (**C**), H_2_O_2_ (**D**), control (**E**), and inlet lake water (**F**)

In terms of molecular size fractions, there was very little statistical difference between any of the treatments. The treatments decreased the fluorescence considerably but did not change the molecular composition in water when compared to the control’s molecular weight profile. However, molecular weight profiles did not stay constant in any of the systems, having minor changes from week to week, but as said earlier, these changes were reflected in all systems at the same time (Fig. 5).

In all cases, the fluorescent DOM was dominantly made of medium-sized molecules (108–1322 Da). There were relatively more small molecules (< 108 Da) than large (> 1322 Da) in DOM, making large molecules the least abundant in water.

There were statistically more medium-sized molecules in high O_3_ treatment than in inlet lake water (*n* = 110, *F* = 5, *p* = 0.028). Control systems also had more small molecules than inlet lake water (*n* = 110, *F* = 5, *p* = 0.020) (Supplementary Table 2). There were only statistical significances found when size fraction data was compared between treatments.

The average molecular weight profile for fluorescence and UV-254 is presented in Supplementary Fig. 3. It is notable that humic and fulvic fluorescence reflected more medium-sized molecules and tyrosine and tryptophan small ones. Tyrosine also had a significant number of large molecules in its signal (*n* = 55, *F* = 4, *p* < 0.05). UV-254 also had more large molecules when compared to humic (*n* = 55, *F* = 4, *p* < 0.05) and fulvic (*n* = 55, *F* = 4, *p* = 0.009) fluorescence (Supplementary Table 5.).

### Fluorescence and UVA-254 – DOC correlation

**Fig. 6:**
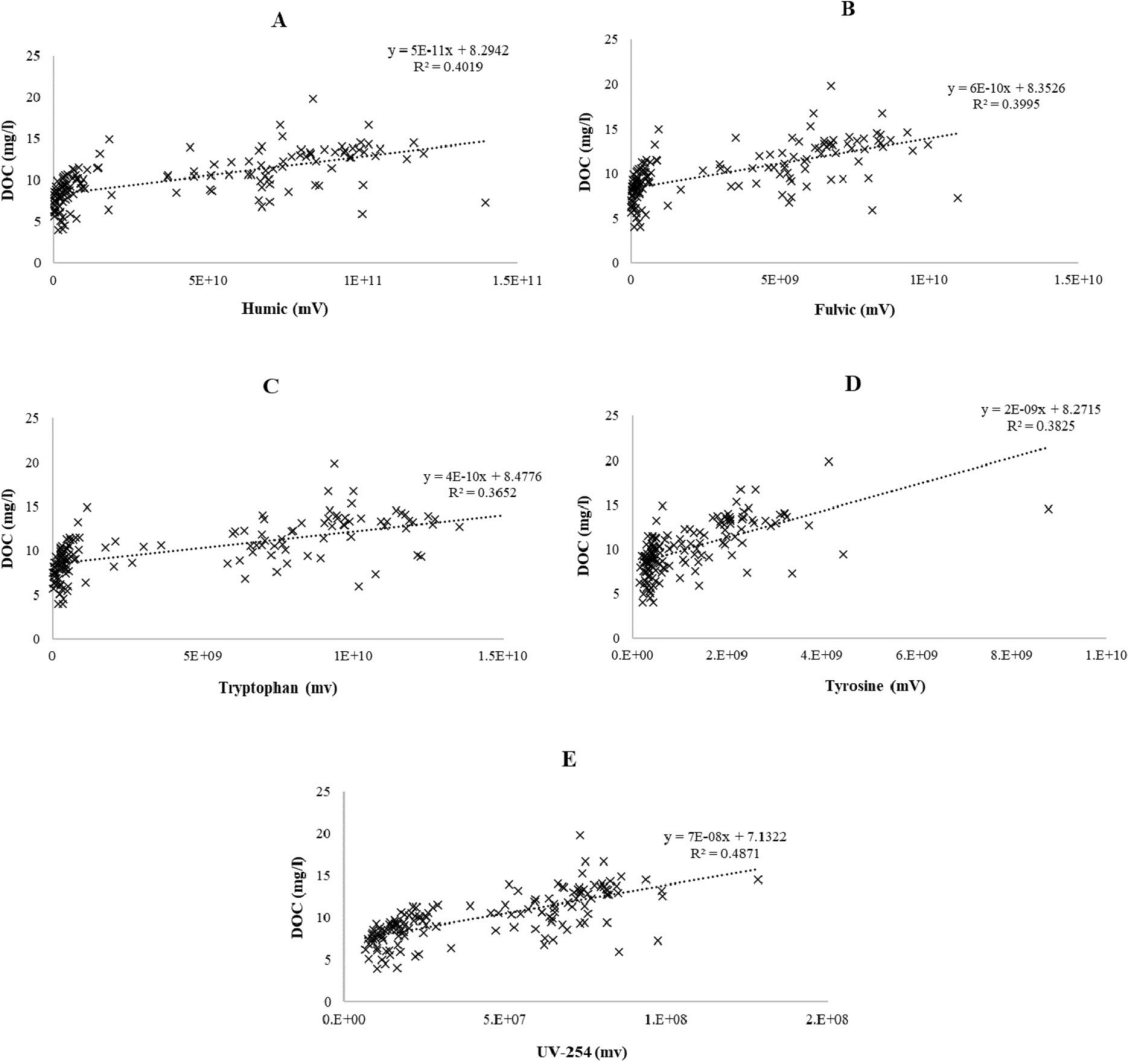
Fluorescence (**A** = humic, **B** = fulvic, **C** = tryptophan, **D** = tyrosine, **E** = UVA-254) as a function of DOC from every system during the whole experiment. Equations and *R*^2^ values for fitted linear models are presented

Figure 6 depicts DOC values plotted against fluorescence responses. Fluorescence gave *R*^2^ values between 0.37 and 0.40. Humic- and fulvic-like fluorescence were on the higher side (around 0.40), as protein-like tryptophan and tyrosine were on the lower side (0.36–0.38). UVA-254 had the best correlation with DOC, *R*^2^ being 0.49 (Fig. 6). Overall, no strong linear relation between fluorescence/UVA-254 and DOC was found.

## Discussion

Initially, all systems started with higher fluorescence and UVA-254 values than inlet lake water. This is to be expected as RAS water is very concentrated with different compounds due to a small water retention rate (Leonard et al., 2002). However, O_3_ and O_3_ + H_2_O_2_ treatments were able to reduce fluorescence to the same or slightly below inlet water levels. The removal of fluorescence was similar to the earlier observations by Spiliotopoulou et al. (2017), with the removal of intensity being in the same range (over 90%). Protein-like fluorescence being removed with worse efficiency than humic-like fluorescence has also been recorded before by Aguilar-Alarcon et al. (2022). The steep decline in fluorescence was followed by a steady residual fluorescence, which indicates that there are always compounds in water that cannot be oxidized by ozone any further or that the reaction rate is so slow that the system can replace them fast enough. In the case that oxidation is limited by ozone’s selectivity, usage of AOP can be beneficial due to the unselectivity of OH radicals (Von Gunten, 2003). However, in this study, AOP treatment was most likely limited by the production of RAS and did not have any advantage over pure O_3_ treatment.

The main part of the DOM in RAS was found to be mainly medium-sized molecules (the main part between 108 and 1322 Da), especially the humic and fulvic parts. Wang et al. (2021) recorded average molecular weights of 400–500 Da with mass spectrometry in their study of aquacultural ponds, and Aguilar-Alarcon et al. (2020) reported similar average molecular weights of 390–450 Da for their research with different feeds in RAS. Our results are in the same size class even though there were substantial differences in environment (the first study had aquacultural ponds, not RAS), water exchange rate, and water quality (the latter study used part seawater).

The molecular weight profile is mostly the same between inlet lake water and RAS systems (except for a few exceptions), which is interesting, as RAS water contains excessive amounts of small nitrogen molecules due to biofiltration and slow water retention rate (Paudel et al., 2015). This, in theory, should make RAS water more concentrated with small molecules when compared to natural water. Ozonation should further decrease the molecule size of DOM in natural waters (Świetlik & Sikorska, 2004; Świetlik et al., 2004; Veenstra et al., 1983), but Krumins et al. (2001) reported that ozonation of RAS does not have any effect to molecular weight profile of DOM due to ozone-induced flocculation and removal by clarifiers. Our results strongly support this theory. In addition, the molecular weight profile in RAS is strongly linked to used inlet water despite the oxidizing treatments.

The protein-like fluorescence tyrosine and tryptophan had a peak in 5 min of retention time, which can be considered as large (40,000–50,000 Da) protein-like structures. These are most likely derived from bacteria, fishes, and feed as they accumulate in system water (Yamin et al., 2017). O_3_ and O_3_ + H_2_O_2_ treatments were able to reduce or keep these compounds stable throughout the experiment, as substantial accumulation can be seen in control and H_2_O_2_ treatments. These fluorescences are considered as indicators of biological activity and bioavailability of DOM (Fellman et al., 2010; Hambly et al., 2015), so their increase in control systems is to be expected as time passes. The protein-like fluorescence being much weaker and different in composition in inlet lake water supports the assumption that most of those molecules are produced in RAS (feed, fish feces, microbial parts) rather than imported from inlet water (Wang et al., 2021; Yamin et al., 2017). This is especially relevant with those large protein-like structures that are almost absent from inlet lake water. On the other hand, humic and fulvic fluorescence share many similarities with RAS, which in turn supports the already existing theory that much of those molecules are generally derived from inlet water in RAS systems (Wang et al., 2021).

Humic and fulvic fluorescence were removed with very similar efficiencies, which somewhat contradicts the earlier reports that ozonation usually targets the large humic-like molecules and supports the formation of smaller fulvic-like molecules (Aguilar-Alarcon et al., 2022; Lai et al., 2021; Veenstra et al., 1983). However, the ozone dose in this study was very large, which can create enough oxidization potential so that the fulvic-like molecules can also be oxidized at the same rate as humic-like molecules.

H_2_O_2_ treatment had very poor fluorescence removal, not being able to remove fluorescence or UVA-254 at all. A better removal efficiency had been expected as the molecular amount of H_2_O_2_ was half of that of low O_3_. H_2_O_2_ is known to be a weaker oxidant than ozone, so the injection amount of oxidizer could have been too low to counter the organic load. There is also a possibility that treatments could boost microbial growth in systems in the same way as ozone transforms large molecules into more bioavailable forms (Wietz et al., 2009). It can also be speculated that Fe^3+^ ions in water can catalyze H_2_O_2_ decomposition if abundant in water (Eisenhauer, 1964). Humic substances can possibly trap this ferric iron to complexes and so reduce their amount in water, which would lead to H_2_O_2_ being more stable. This is all just speculation, and no evidence can be presented here, but it could explain the difference among systems as such.

H_2_O_2_ underwhelming fluorescence reduction indicates that something might be happening outside of monitoring wavelengths, and so it went unrecorded. In Pettersson et al. (2022), fishes in H_2_O_2_ treatment had improved growth, so water quality indeed seemed to improve in some areas. The slight reduction in tryptophan and tyrosine peaks when compared to control could display disinfection happening because of less microbial activity. Turbidity was lower in H_2_O_2_ than in control systems but higher than in other treatments (Supplementary Table 3). However, this reduction in turbidity was not noticeable in fluorescence, probably mostly as filtration before HPLSEC analysis removed all the suspended solids, colloids, etc.

The least efficient UVA-254 removal was possibly caused by aromatic rings. These rings are one of the harder things for ozone to oxidize, and UVA-254, being a meter for DOM aromaticity, could explain these results. UVA-254 also represents double and triple bonds, which will be oxidized immediately by ozone, but it is possible that these are in the minority when compared to aromatic rings. The less selective hydroxyl radical from O_3_ + H_2_O_2_ treatment should react with aromatic rings better than pure ozone (Von Gunten, 2003), but this was not observed here. The RAS water is heavily concentrated with different compounds, which simultaneously contribute to the fluorescence and UVA and thus cannot be monitored individually. It is possible that hydroxyl radicals created in the O_3_ + H_2_O_2_ process are more likely to be scavenged by these compounds than ozone, resulting in observed similar efficiency among pure O_3_ and O_3_ + H_2_O_2_ treatments (Klausen & Gronborg, 2010). Again, it seems that the possible advantages of AOP are limited by DOM concentrations.

The substantial UVA-254 peak of approximately 12 min of retention time is curious, as in that molecular weight range, only benzoic acid and its derivatives can be found. Benzoic acid has been reported to be found in a few different RAS systems (Lindholm-Lehto et al., 2020, 2021), and it is produced by micro-organisms, plants, algae, and animals in their cells as part of their metabolism (Qualley et al., 2012; Joye, 2019). The peak, however, is fairly strong and temporarily growing in relation to other peaks, which indicates that molecules represented by it are relatively abundant. One possibility is that treatments are cutting big aromatic compounds with multiple rings into smaller pieces (possibly attacking the before-mentioned double and triple bonds), which then leads finally to benzoic acid-like compounds. This would explain the relative growth of the peak.

The O_3_ had a clear reducing effect on DOC concentrations, and that was to be expected from previous studies (Park et al., 2013; Summerfelt et al., 1997). The reduction in DOC was higher in this study than in those referenced ones, but so was the ozone dose, which most probably explains the difference. Interestingly, the O_3_ + H_2_O_2_ treatment had the same DOC concentrations as the low O_3_ treatment. Both treatments had the same amount of ozone injected into water, which means that O_3_ + H_2_O_2_’s oxidative potential should have been higher and possibly lead to lower DOC concentrations. The high O_3_ treatment had the lowest concentrations of DOC, which indicates that by increasing the ozone injection to the system, the DOC can be further decreased, but the efficiency will suffer as compounds that are harder to oxidize become more abundant.

There is a significant difference in DOC concentration between O_3_ + H_2_O_2_ and high O_3_. On the other hand, the fluorescence difference between O_3_ + H_2_O_2_ and high O_3_ is very small and not notable, so clearly, this missing DOC is something that cannot be seen in the fluorescence measurements. If trusted purely on fluorescence, it could be said that doubling the ozone dose is just a waste of resources as the payoff is nonexistent, but this would then completely ignore the overall DOC reduction if the same comparison is done to O_3_ + H_2_O_2_ and low O_3_, fluorescence, and DOC line logically.

No strong linear correlation between fluorescence and DOC was observed in this experiment. UVA-254 had the strongest relation, which is not surprising as highly aromatic humic and fulvic acids represent up to 80% of DOM in natural waters (Singh, 2014). UVA-254 is already regularly used as an indicator for organic matter in aquaculture. Ignatev and Tuhkanen (2019) proposed that fluorescence and DOC would have a linear relation that could be used in predicting organic matter in water. It worked well in the case of wastewater purification, but it seems clear that ozonation changes the organic matter structure in RAS water in a way that at least the fluorescence HPLSEC method used here cannot predict the DOC content of water reliably. It could be speculated that in RAS, some fluorescence compounds are formed upon ozonation that emit strong fluorescence signals but are low in concentration. There is no evidence to support this, but it can be one way to explain this phenomenon.

## Conclusions

Significant differences in chromatograms were found among the treatments and inlet lake water. Mainly, the intensity was much higher in RAS water when compared to inlet water. O_3_ and O_3_ + H_2_O_2_ treatments greatly reduced fluorescence, even close to inlet water fluorescence values, while H_2_O_2_ had no detectable effect. O_3_ + H_2_O_2_ treatment added no benefits over normal O_3_ treatment, which indicated that oxidation of DOM was not limited by the selectivity of ozone but rather the organic loading of the systems. Fluorescence demonstrated that most of the humic and fulvic compounds were derived from the inlet water, while protein-like fluorescence, especially the large molecules, is produced in RAS. UV-254 showed that RAS water contained large amounts of small aromatic benzoic acid derivates that were not detected in inlet lake water. The studied fluorescence HPLSEC method proved to be sensitive enough to track the oxidizer-induced change in DOM in the RAS environment.

Most of the fluorescence intensity was produced by medium-sized molecules (108–1322 Da). This was true for both RAS and inlet lake water, despite RAS being usually reported to contain smaller molecules in comparison. Treatments also did not have any effect on the molecular weight profile of the water. This proves that even with strong oxidizing treatments, the molecular weight profile of RAS water is strongly linked to its water source.

The O_3_ was able to substantially reduce the DOC of RAS water. The deduced relation between fluorescence and DOC is not a valid meter in the RAS environment if water is treated with continuous ozonation. This, however, does not mean that this relation should not be studied further with non-ozonated RAS waters, as the method has worked well in different environments.

### Supplementary Information

Below is the link to the electronic supplementary material.

Supplementary file1 (DOCX 475 KB)

## Data Availability

All data used in this manuscript can be obtained by emailing the corresponding author.
