# Supplementary material for: Monitoring of water quality with HPLSEC and fluorescence method in the ozonated recirculating aquaculture system
Source: Environ Monit Assess. 2023 Nov 20;195(12):1497. doi: 10.1007/s10661-023-12117-5 (PMC10661748; doi:10.1007/s10661-023-12117-5)
Supplement: Supplementary file 1 — Supplementary file1 (DOCX 475 KB) [file 10661_2023_12117_MOESM1_ESM.docx]

# Supplementary material

Supplementary Table 1. Kruskal-Wallis comparison between the treatments and inlet water for fluorescence and UV-254. Sample size (n), degree of freedom (F), average difference between treatments (test statistic) and significance (p) are presented. Significance 0.000 = <0.000.

| **Fluorescence** | **Comparison** | **n** | **F** | **Test statistic** | ***p*** |
| --- | --- | --- | --- | --- | --- |
| **Tryptophan** | O_3_HIGH-O_3_LOW | 132 | 5 | 19 | 0.086 |
|  | O_3_HIGH-O_3_+H_2_O_2_ |  |  | 29 | 0.008 |
|  | O_3_HIGH-INLET |  |  | -55 | 0.000 |
|  | O_3_HIGH-H_2_O_2_ |  |  | -84 | 0.000 |
|  | O_3_HIGH-CONTROL | |  | -91 | 0.000 |
|  | O_3_LOW- O_3_+H_2_O_2_ |  |  | 10 | 0.351 |
|  | O_3_LOW-INLET |  |  | -36 | 0.008 |
|  | O_3_LOW-H_2_O_2_ |  |  | -65 | 0.000 |
|  | O_3_LOW-CONTROL | |  | -72 | 0.000 |
|  | O_3_+H_2_O_2_-INLET |  |  | -26 | 0.059 |
|  | O_3_+H_2_O_2_-H_2_O_2_ |  |  | -55 | 0.000 |
|  | O_3_+H_2_O_2_-CONTROL |  |  | -62 | 0.000 |
|  | INLET-H_2_O_2_ |  |  | 29 | 0.031 |
|  | INLET-CONTROL | |  | 37 | 0.007 |
|  | H_2_O_2_-CONTROL | |  | 7 | 0.507 |
| **Tyrosin** | INLET-O_3_HIGH | 132 | 5 | 3 | 0.827 |
|  | INLET-O_3_LOW |  |  | 11 | 0.433 |
|  | INLET- O_3_+H_2_O_2_ |  |  | 20 | 0.135 |
|  | INLET-H_2_O_2_ |  |  | 71 | 0.000 |
|  | INLET-CONTROL | |  | 80 | 0.000 |
|  | O_3_HIGH-O_3_LOW | |  | 8 | 0.489 |
|  | O_3_HIGH- O_3_+H_2_O_2_ |  |  | 17 | 0.118 |
|  | O_3_HIGH-H_2_O_2_ |  |  | -68 | 0.000 |
|  | O_3_HIGH-CONTROL | |  | -77 | 0.000 |
|  | O_3_LOW- O_3_+H_2_O_2_ |  |  | 10 | 0.383 |
|  | O_3_LOW-H_2_O_2_ |  |  | -61 | 0.000 |
|  | O_3_LOW-CONTROL | |  | -69 | 0.000 |
|  | O_3_+H_2_O_2_-H_2_O_2_ |  |  | -51 | 0.000 |
|  | O_3_+H_2_O_2_-CONTROL |  |  | -59 | 0.000 |
|  | H_2_O_2_-CONTROL | |  | 8 | 0.450 |
| **Humic** | O_3_HIGH-O_3_LOW | 132 | 5 | 23 | 0.034 |
|  | O_3_HIGH- O_3_+H_2_O_2_ |  |  | 31 | 0.004 |
|  | O_3_HIGH-INLET |  |  | -54 | 0.000 |
|  | O_3_HIGH-CONTROL | |  | -87 | 0.000 |
|  | O_3_HIGH-H_2_O_2_ |  |  | -92 | 0.000 |
|  | O_3_LOW- O_3_+H_2_O_2_ |  |  | 8 | 0.471 |
|  | O_3_LOW-INLET |  |  | -31 | 0.023 |
|  | O_3_LOW-CONTROL | |  | -64 | 0.000 |
|  | O_3_LOW-H_2_O_2_ |  |  | -68 | 0.000 |
|  | O_3_+H_2_O_2_-INLET |  |  | -23 | 0.093 |
|  | O_3_+H_2_O_2_-CONTROL |  |  | -56 | 0.000 |
|  | O_3_+H_2_O_2_-H_2_O_2_ |  |  | -60 | 0.000 |
|  | INLET-CONTROL | |  | 33 | 0.015 |
|  | INLET-H_2_O_2_ |  |  | 38 | 0.006 |
|  | CONTROL-H_2_O_2_ | |  | -5 | 0.678 |
| **Fulvic** | O_3_HIGH-O_3_LOW | 132 | 5 | 22 | 0.048 |
|  | O_3_HIGH- O_3_+H_2_O_2_ |  |  | 30 | 0.007 |
|  | O_3_HIGH-INLET |  |  | -57 | 0.000 |
|  | O_3_HIGH-CONTROL | |  | -86 | 0.000 |
|  | O_3_HIGH-H_2_O_2_ |  |  | -92 | 0.000 |
|  | O_3_LOW-O3+H2O2 |  |  | 8 | 0.466 |
|  | O_3_LOW-INLET |  |  | -36 | 0.009 |
|  | O_3_LOW-CONTROL | |  | -64 | 0.000 |
|  | O_3_LOW-H_2_O_2_ |  |  | -70 | 0.000 |
|  | O_3_+H_2_O_2_-INLET |  |  | -28 | 0.042 |
|  | O_3_+H_2_O_2_-CONTROL |  |  | -56 | 0.000 |
|  | O_3_+H_2_O_2_-H_2_O_2_ |  |  | -62 | 0.000 |
|  | INLET-CONTROL | |  | 29 | 0.034 |
|  | INLET-H_2_O_2_ |  |  | 34 | 0.011 |
|  | CONTROL-H_2_O_2_ | |  | -6 | 0.608 |
| **UV-254** | O_3_HIGH-O_3_LOW | 132 | 5 | 24 | 0.033 |
|  | O_3_HIGH- O_3_+H_2_O_2_ |  |  | 34 | 0.002 |
|  | O_3_HIGH-INLET |  |  | -51 | 0.000 |
|  | O_3_HIGH-CONTROL | |  | -85 | 0.000 |
|  | O_3_HIGH-H_2_O_2_ |  |  | -90 | 0.000 |
|  | O_3_LOW- O_3_+H_2_O_2_ |  |  | 10 | 0.355 |
|  | O_3_LOW-INLET |  |  | -28 | 0.040 |
|  | O_3_LOW-CONTROL | |  | -61 | 0.000 |
|  | O_3_LOW-H_2_O_2_ |  |  | -67 | 0.000 |
|  | O_3_+H_2_O_2_-INLET |  |  | -18 | 0.194 |
|  | O_3_+H_2_O_2_-CONTROL |  |  | -51 | 0.000 |
|  | O_3_+H_2_O_2_-H_2_O_2_ |  |  | -56 | 0.000 |
|  | INLET-CONTROL | |  | 33 | 0.014 |
|  | INLET-H_2_O_2_ |  |  | 39 | 0.004 |
|  | CONTROL-H_2_O_2_ | |  | -6 | 0.605 |
|  |  | |  |  |  |

Supplementary Table 2. Kruskal-Wallis comparison between treatments for different size fractions. Sample size (n), degree of freedom (F), average difference between treatments (test statistic) and significance (p) are presented. Significance 0.000 = <0.000.

| **Fraction** | **Comparison** | **n** | **F** | **Test statistic** | **sig.** |
| --- | --- | --- | --- | --- | --- |
| **Large** | Inlet-Low O_3_ | 110 | 5 | 0.2 | 0.987 |
|  | Inlet-O_3_+H_2_O_2_ |  |  | 2.6 | 0.833 |
|  | Inlet-High O_3_ |  |  | 4.1 | 0.774 |
|  | Inlet-Control |  |  | 9.8 | 0.399 |
|  | Inlet-H_2_O_2_ |  |  | 18.4 | 0.137 |
|  | Low O_3_-O_3_+H_2_O_2_ |  |  | 2.4 | 0.812 |
|  | Low O_3_-High O_3_ |  |  | -3.9 | 0.752 |
|  | Low O_3_-Control |  |  | -9.7 | 0.295 |
|  | Low O_3_-H_2_O_2_ |  |  | -18.2 | 0.072 |
|  | O_3_+H_2_O_2_-High O_3_ |  |  | -1.5 | 0.903 |
|  | O_3_+H_2_O_2_-Control |  |  | -7.2 | 0.432 |
|  | O_3_+H_2_O_2_-H_2_O_2_ |  |  | -15.8 | 0.118 |
|  | High O_3_-Control |  |  | -5.8 | 0.623 |
|  | High O_3_-H_2_O_2_ |  |  | -14.3 | 0.249 |
|  | Control-H_2_O_2_ |  |  | -8.6 | 0.355 |
| **Medium** | High O_3_-Control | 110 | 5 | -5.1 | 0.659 |
|  | High O_3_-O_3_+H_2_O_2_ |  |  | 7.3 | 0.557 |
|  | High O_3_- H_2_O_2_ |  |  | -14.3 | 0.247 |
|  | High O_3_-Low O_3_ |  |  | 16.7 | 0.176 |
|  | High O_3_-Inlet |  |  | -31.3 | 0.028 |
|  | Control-O_3_+H_2_O_2_ |  |  | 2.1 | 0.818 |
|  | Control- H_2_O_2_ |  |  | -9.2 | 0.319 |
|  | Control-Low O_3_ |  |  | 11.7 | 0.209 |
|  | Control-Inlet |  |  | -26.2 | 0.024 |
|  | O_3_+H_2_O_2_-H_2_O_2_ |  |  | -7.1 | 0.484 |
|  | O_3_+H_2_O_2_-Low O_3_ |  |  | -9.5 | 0.348 |
|  | O_3_+H_2_O_2_-Inlet |  |  | -24.1 | 0.052 |
|  | H_2_O_2_-Low O_3_ |  |  | 2.4 | 0.811 |
|  | H_2_O_2_-Inlet |  |  | -17 | 0.168 |
|  | Low O_3_-Inlet |  |  | -14.6 | 0.237 |
| **Small** | Inlet- H_2_O_2_ | 110 | 5 | 12.8 | 0.300 |
|  | Inlet-O_3_+H_2_O_2_ |  |  | 14.9 | 0.229 |
|  | Inlet-Low O_3_ |  |  | 17.8 | 0.151 |
|  | Inlet-Control |  |  | 27.1 | 0.020 |
|  | Inlet-High O_3_ |  |  | 34.7 | 0.015 |
|  | H_2_O_2_-O_3_+H_2_O_2_ |  |  | 2.1 | 0.839 |
|  | H_2_O_2_-Low O_3_ |  |  | 5.0 | 0.624 |
|  | H_2_O_2_-Control |  |  | 14.3 | 0.120 |
|  | H_2_O_2_-High O_3_ |  |  | 21.9 | 0.076 |
|  | O_3_+H_2_O_2_-Low O_3_ |  |  | -2.9 | 0.774 |
|  | O_3_+H_2_O_2_-Control |  |  | -12.3 | 0.183 |
|  | O_3_+H_2_O_2_-High O3 |  |  | -19.9 | 0.108 |
|  | Low O_3_-Control |  |  | -9.4 | 0.310 |
|  | Low O_3_-High O_3_ |  |  | -17.0 | 0.170 |
|  | Control-High O_3_ |  |  | 7.6 | 0.514 |

Supplementary Table 3. Water turbidity measured and averaged for all treatments, from every week.

| **Week** | **Control** | **H_2_O_2_** | **Low O_3_** | **High O_3_** | **O_3_+H_2_O_2_** |
| --- | --- | --- | --- | --- | --- |
| 2 | 1.8 | 1.8 | 2.2 | 2.2 | 2.4 |
| 3 | 1.9 | 1.1 | 1.4 | 1.2 | 1.2 |
| 4 | 3.3 | 2.2 | 4.8 | 1.3 | 1.3 |
| 5 | 7.3 | 3.7 | 1.1 | 1.1 | 2.5 |
| 6 | 11.4 | 4.3 | 1.5 | 2.2 | 1.2 |
| 7 | 7.7 | 5.8 | 1.6 | 1.9 | 1.6 |
| 8 | 6.2 | 5.0 | 1.1 | 1.6 | 1.0 |
| 9 | 6.2 | 2.1 | 1.1 | 2.0 | 1.3 |
| 10 | 6.9 | 3.0 | 1.4 | 2.0 | 1.0 |
| 11 | 7.1 | 3.0 | 1.5 | 2.1 | 1.4 |
| 12 | 5.6 | 2.7 | 1.4 | 2.1 | 1.3 |
| 13 | 5.5 | 3.6 | 1.7 | 1.7 | 1.7 |
| 14 | 3.6 | 2.6 | 1.3 | 1.9 | 1.2 |
| 15 | 5.8 | 1.7 | 1.4 | 1.6 | 1.5 |
|  |  |  |  |  |  |

Supplementary Table 4. Results from statistical analyzes for DOC. Used significance level was 0.05. Significance 0.000 = <0.000.

|  | **Comparison** | **n** | **Degree of freedom** | **Sig.** | **Test**  **Statistic** |
| --- | --- | --- | --- | --- | --- |
| **DOC** | High O_3_-Low O_3_ | 28 | 4 | 0.063 | 0.79 |
|  | High O_3_- O_3_+H_2_O_2_ |  |  | 0.035 | 0.89 |
|  | High O_3_-H_2_O_2_ |  |  | 0.000 | -2.25 |
|  | High O_3_-Control |  |  | 0.000 | 2.32 |
|  | Low O_3_- O_3_+H_2_O_2_ |  |  | 0.800 | 0.11 |
|  | Low O_3_-H_2_O_2_ |  |  | 0.001 | -1.46 |
|  | Low O_3_-Control |  |  | 0.000 | -1.54 |
|  | O_3_+H_2_O_2_-H_2_O_2_ |  |  | 0.001 | -1.36 |
|  | O_3_+H_2_O_2_-Control |  |  | 0.001 | -1.43 |
|  | H_2_O_2_-Control |  |  | 0.866 | 0.07 |

Supplementary figure 1. Standard line for molecular weight (Da) and retention time (min). Displaying the equation and R^2^-value.


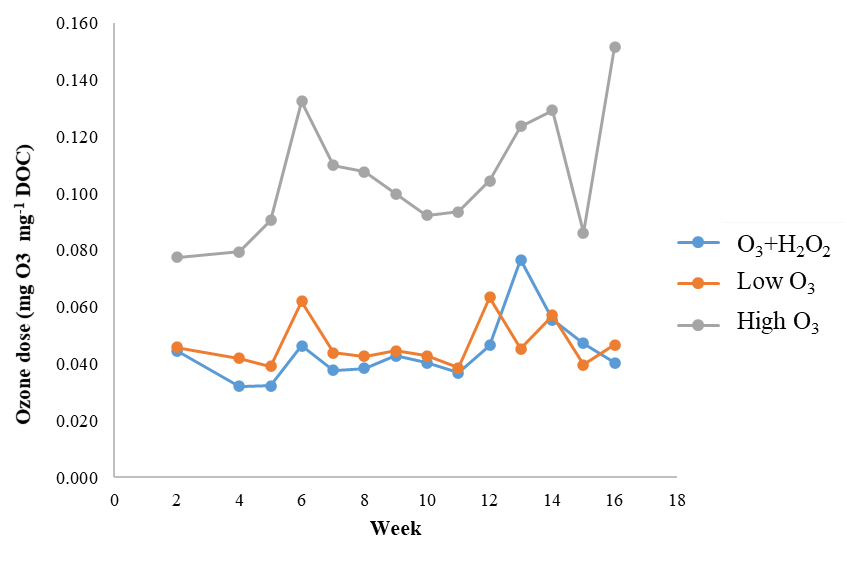


Supplementary figure 2. Calculated Ozone doses (mg O3/ mg DOC) applied in systems treated with ozone.

Supplementary Table 5. Kruskal-Wallis comparison between fluorescence and UV-254 for different size fractions. Sample size (n), degree of freedom (F), average difference between treatments (test statistic) and significance (p) are presented. Significance 0.000 = <0

| **Fractrion** | **Comparison** | **n** | **F** | **Test statistic** | **sig.** |
| --- | --- | --- | --- | --- | --- |
| **Large** | Humic-Tryptophan | 55 | 4 | -8.3 | 0.226 |
|  | Humic-Fulvic |  |  | -10.3 | 0.133 |
|  | Humic-UV-254 |  |  | -28.0 | 0.000 |
|  | Humic-Tyrosine |  |  | -32.5 | 0.000 |
|  | Tryptophan-Fulvic |  |  | 2.0 | 0.770 |
|  | Tryptophan-UV-254 |  |  | -19.7 | 0.004 |
|  | Tryptophan-Tyrosine |  |  | 24.3 | 0.000 |
|  | Fulvic-UV-254 |  |  | -17.7 | 0.009 |
|  | Fulvic-Tyrosine |  |  | -22.3 | 0.001 |
|  | UV-254-Tyrosine |  |  | 4.5 | 0.506 |
|  | Humic-Tryptophan |  |  | -8.3 | 0.226 |
|  | Humic-Fulvic |  |  | -10.3 | 0.133 |
|  | Humic-UV-254 |  |  | -28.0 | 0.000 |
|  | Humic-Tyrosine |  |  | -32.5 | 0.000 |
|  | Tryptophan-Fulvic |  |  | 2.0 | 0.770 |
| **Medium** | Tyrosine-Tryptophan | 55 | 4 | -12.5 | 0.068 |
|  | Tyrosine-UV-254 |  |  | -24.2 | 0.000 |
|  | Tyrosine-Fulvic |  |  | 32.1 | 0.000 |
|  | Tyrosine-Humic |  |  | 41.3 | 0.000 |
|  | Tryptophan-UV-254 |  |  | -11.7 | 0.086 |
|  | Tryptophan-Fulvic |  |  | 19.6 | 0.004 |
|  | Tryptophan-Humic |  |  | 28.8 | 0.000 |
|  | UV-254-Fulvic |  |  | 7.9 | 0.247 |
|  | UV-254-Humic |  |  | 17.1 | 0.012 |
|  | Tyrosine-Tryptophan |  |  | 9.2 | 0.179 |
|  | Tyrosine-UV-254 |  |  | -12.5 | 0.068 |
|  | Tyrosine-Fulvic |  |  | -24.2 | 0.000 |
|  | Tyrosine-Humic |  |  | 32.1 | 0.000 |
|  | Tryptophan-UV-254 |  |  | 41.3 | 0.000 |
|  | Fulvic-Humic |  |  | -11.7 | 0.086 |
| **Small** | UV-254-Humic | 55 | 4 | 6.182 | 0.366 |
|  | UV-254-Fulvic |  |  | 9.182 | 0.179 |
|  | UV-254-Tryptophan |  |  | 26.818 | 0.000 |
|  | UV-254-Tyrosine |  |  | 35.545 | 0.000 |
|  | Humic-Fulvic |  |  | -3.000 | 0.661 |
|  | Humic-Tryptophan |  |  | -20.636 | 0.003 |
|  | Humic-Tyrosine |  |  | -29.364 | 0.000 |
|  | Fulvic-Tryptophan |  |  | -17.636 | 0.010 |
|  | Fulvic-Tyrosine |  |  | -26.364 | 0.000 |
|  | UV-254-Humic |  |  | 8.727 | 0.201 |
|  | UV-254-Fulvic |  |  | 6.182 | 0.366 |
|  | UV-254-Tryptophan |  |  | 9.182 | 0.179 |
|  | UV-254-Tyrosine |  |  | 26.818 | 0.000 |
|  | Humic-Fulvic |  |  | 35.545 | 0.000 |
|  | Tryptophan-Tyrosine |  |  | -3.000 | 0.661 |

Supplementary Figure 3. Average percentual distribution of molecule size fractions among the fluorescence (A=Humic, B=Fulvic, C=Tyrosine, D=Tryptophan) and UVA-254 (E).


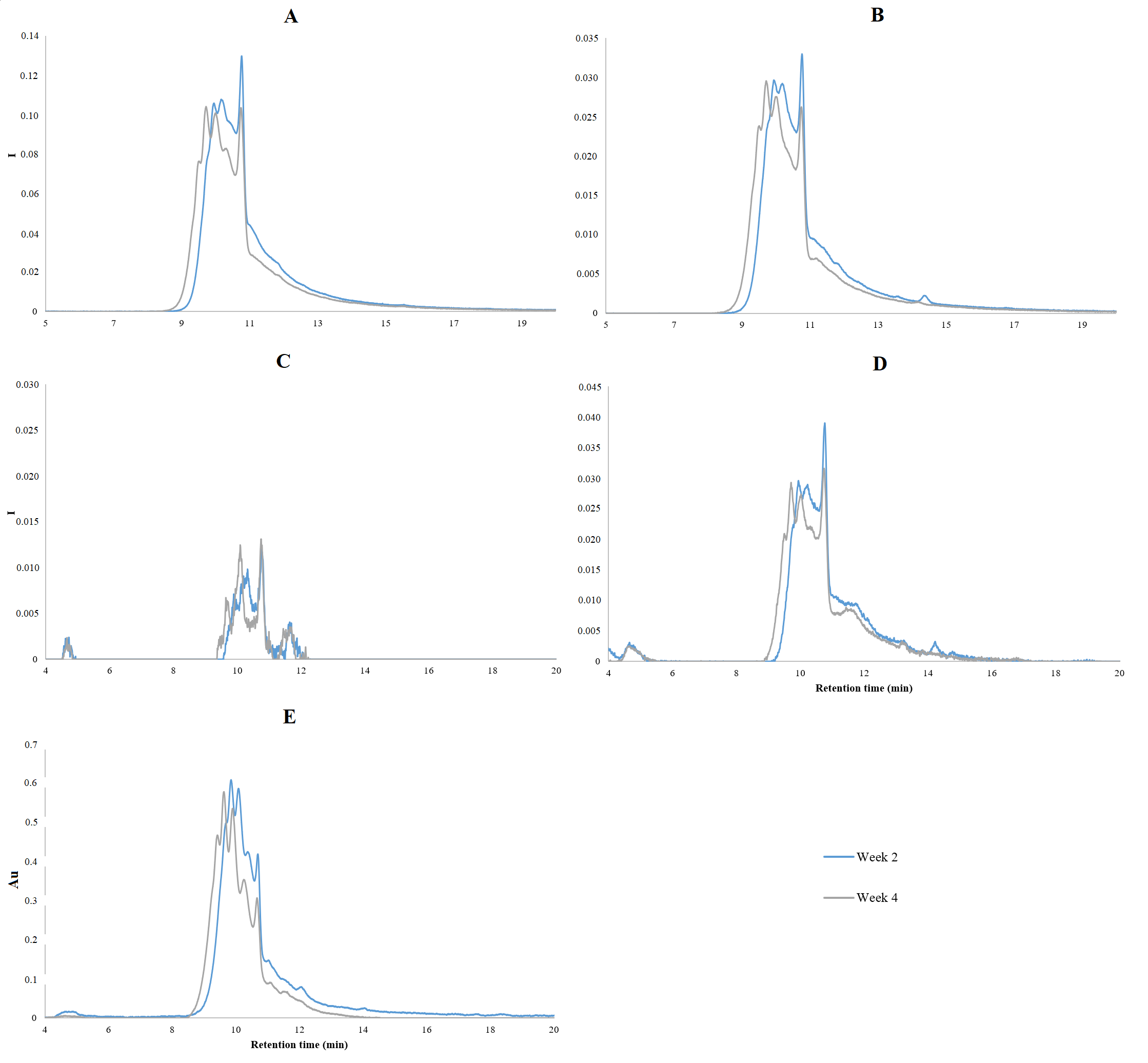


Supplementary figure 4. Chromatograms for Inlet lake water from weeks 2 and 4. Fluorescences are A=Humic, B= Fulvic, C=Tyrosin, D=Tryptophan and E= UVA-254.
